# Key Factors Influencing the Adoption of CAD/CAM in the Fabrication of Indirect Dental Restorations

**DOI:** 10.1155/ijod/4948410

**Published:** 2026-04-13

**Authors:** Ana Kahurangi Conway Downey, Vincent Bennani, Arthi Veerasamy

**Affiliations:** ^1^ Sir John Walsh Research Institute, University of Otago, Dunedin, New Zealand, otago.ac.nz; ^2^ Department of Oral Rehabilitation, University of Otago, Dunedin, New Zealand, otago.ac.nz

**Keywords:** CAD/CAM, CEREC, dental technicians, dentists, digital technologies

## Abstract

**Introduction:**

Over recent decades, CAD/CAM (computer assisted design/computer assisted manufacture) has become increasingly prevalent in dentistry. These systems offer alternatives to various stages of the conventional workflow for indirect restorations. While CAD/CAM has been explored in previous studies, their use in New Zealand (NZ) remains under‐researched. This study aimed to assess the prevalence of CAD/CAM usage and identify factors influencing their adoption among dentists and dental technicians.

**Methods:**

Questionnaires adapted from previous research were distributed to 1703 registered dentists and 198 dental technicians via the DCNZ registry. Responses were analysed descriptively, with chi‐square and Fisher’s exact tests used to examine associations.

**Results:**

A total of 332 dentists responded (19% response rate) and 40 dental technicians responded. CAD/CAM was used by 59% of dentists and 87% of technicians for indirect restorations. Intraoral scanning and chairside systems were the most common applications, used by 93% and 66% of CAD/CAM users, respectively. Key barriers included cost and lack of training; primary facilitators were increased efficiency and staying current with technology. Gender, practice typeand volume of restorations were significantly associated with adoption among dentists.

**Conclusion:**

This study’s findings highlight the growing prominence of CAD/CAM in dentistry, with dental technicians adopting them more rapidly than dentists. To close this gap, enhancements in dental education and targeted training programmes are crucial for supporting and accelerating the integration of digital workflows into clinical practice.

## 1. Introduction

Indirect restorations can be an effective route for achieving patient satisfaction by improving aesthetics and functionality [1]. Crowns, bridges, veneers, onlays and inlays are used to rehabilitate lost tooth structure and/or missing teeth [2]. Two significant components of the development of indirect restorations are the use of CAD/CAM (computer assisted design/computer assisted manufacture) and the role of dental technicians [3].

Indirect restorations can be produced through fully digital or conventional workflows. Hybrid workflows involve both digital and conventional steps. Conventional workflows involve the use of impressions, which are then taken to dental laboratories to manufacture indirect restorations through manual techniques such as lost wax casting, slip casting, heat pressing and powder condensation [4–7]. Digital workflows involve the use of CAD/CAM. In this study, ’digital workflow’ refers broadly to workflows involving CAD/CAM technologies, including intraoral scanning, digital design and computer‐assisted manufacturing.

The three stages of the digital workflow are data acquisition, digital design and fabrication [8]. For data acquisition, intra‐oral scanners can be used to record information directly from the mouth. Alternatively, extra‐oral/laboratory scanners can scan models or conventional impressions. Many types of software have been developed for CAD, such as Exocad and 3Shape. These designs can then be used for CAM, which can involve additive processes such as 3D printing and laser sintering, or subtractive processes such as NewZealand (NZ) European decentmilling [9–11]. Dental technicians play a prominent role in dictating the types of technology and equipment used for design and manufacturing [12]. Technologies like CEREC allow dentists to carry out fully digital, chairside workflows without the input of dental technicians. In 2015 and 2016, between 20% and 32% of NZ dentists had access to chairside CAD/CAM machines [9, 13].

Previous research in NZ has investigated the factors that influence the adoption of newer technologies, including one type of CAD/CAM, which was the chairside restoration system CEREC. The most important factors were improving the quality of care, efficiency, ease of use and adding treatment options. Less important factors included improved communication, ease of storage and environmental concerns [13]. These results are consistent with international evidence [14, 15].

There is little NZ research on the communication between dentists and dental technicians and the influence of digital technologies. Overseas research, however, consistently finds that a significant proportion of prescriptions provide insufficient information and frequently require extra communication to compensate for gaps [16, 17]. Dentists may do this to avoid planning and rely on technicians to make certain decisions [12].

NZ data on the use of CAD/CAM by dental technicians differs greatly to international equivalents and is outdated. As of 2012, a quarter of NZ dental laboratories owned CAD systems, and 7.9% had full CAD/CAM systems [18]. In laboratories from the United States (US) in 2020 70% of cases involved hybrid or fully digital workflows [19]. This disparity could be due to geographic variation or the time lapse between these studies. Updated data is necessary to assess whether NZ uptake is still lagging behind international equivalents.

This study aimed to update existing data and examine how the prominence of CAD/CAM technology has evolved among dental technicians and dentists. Given that previous research in NZ has primarily focused on chairside applications, this study also sought to take a more holistic approach, exploring a broader range of CAD/CAM uses. Additionally, it investigated the factors influencing the adoption of digital technologies and how collaboration between dentists and dental technicians impacts this process.

## 2. Materials and Methods

To investigate the factors influencing the adoption of digital technologies in the manufacture of indirect restorations, two questionnaires, one for dentists and one for dental technicians, were adapted from previous studies [14, 16, 18, 20]. There were 28 questions for dentists and 25 questions for dental technicians. There were six sections, and all participants were shown the first and the last. Participants were shown one or two other sections depending on their CAD/CAM usage. Most questions were multiple choice and allowed more than one answer. Some questions asked for a short, written answer including gender, year of qualification and commonly used materials. Both surveys included questions on participant demographics, material selection, reasons for and again adoption, types of CAD/CAM used and prescription quality.

Ethical approval was obtained through the University of Otago Human Ethics Committee (25/0842). Māori consultation was also done through the Ngāi Tahu Research Consultation Committee. Both surveys were tested out on pilot studies of 10 people before the questions were refined. To be included in the study, dentists had to be registered in NZ with an annual practising certificate and have fabricated an indirect restoration within the last 12 months. To be included, dental technicians needed to be working in NZ in either a commercial laboratory, an inhouse practice laboratory, or academic institution and fabricate at least one indirect restoration a month. The dentist survey was sent to the 2025 DCNZ list of registered dentists with a current annual practising certificate, who had indicated they were open to receiving invitations for research, which included 1703 dentists. However, the DCNZ workforce data suggested that 3300 practising dentists are in NZ. With our sample of 332 dentists, the 95% confidence interval for a proportion near 50% is ±2.7% (slightly narrower after applying finite population correction. Because our sample represented about 10% of all practising dentists in NZ, the finite population correction slightly reduced the margin of error.

The dental technician questionnaire was sent to the DCNZ list of registered dental technicians, including 198 technicians. As NZ dental technicians do not need to be registered, 26 dental laboratories were also invited via email or phone call. Questionnaires were accessed through Qualtrix^XM^ and distributed via email. Two reminder emails were sent out. Participants read the participant information sheet and consent form and gave informed consent.

Descriptive statistics were performed for both questionnaires. Where appropriate, proportions were interpreted with consideration of sampling variability. Bivariate analysis was completed for the dentist survey using chi‐square tests, Fisher’s exact tests and Kruskal–Wallis tests to investigate the association between CAD and CAM usage with factors such as gender, years since graduation, practice type, ethnicity, region and number of indirect restorations completed per week. SPSS (SPSS v29.0, IBM Corp., Armonk, NY, USA) software was used to process the data.

## 3. Results

Three hundred and thirty‐two dentists responded to the questionnaire (response rate 19%). Participant characteristics are summarised in Table 1. Respondents were distributed across all regions of NZ, suggesting broad geographic representation of the dental workforce. Participants were recorded from all regions of NZ; however, Auckland was the most common at 33%. Most participants were of NZ European descent (53%) and working as general dental practitioners (83%). Dentists from small (50%) or large (43%) group practices made up the majority and working at franchises (9%) and in the public system (7%) were much more uncommon.

**Table 1:** Demographics of dentists.

Option	Count	Percentage (%)
Which race/ethnicity best describes you (please select all that apply)		
New Zealand European	164	53
Māori	12	4
Samoan	3	1
Chinese	49	16
Indian	30	10
Other	71	23

Please choose your work location based on region? (Please select all that apply)		
Northland	10	3
Auckland	103	33
Waikato	24	8
Bay of Plenty	17	5
Gisborne	2	1
Manawatū‐Whanganui	10	3
Hawkes Bay	11	4
Taranaki	4	1
Wellington	43	14
Nelson–Marlborough	15	5
West Coast	2	1
Canterbury	36	12
Otago	32	10
Southland	7	2

How many fixed prostheses (such as single‐unit crowns, fixed partial dentures, bridges or onlays) do you do each month in your practice?		
0	13	4
1–5	124	40
6–10	88	28
10–15	46	15
>16	39	13

How much formal training have you had?		
GDP	259	83
Postgraduate training	46	15
Other	7	2

Where do you work? (Please tick all that apply)		
Solo or small group private practice (fewer than three practising dentists)	156	50
Large group practice	136	43
Franchise	28	9
Public health practice like hospital	22	7

Do you use any aspect of CAD/CAM in your workflow?		
Yes	184	59
No	98	31
Have used in the past but no longer use currently	30	10

Forty dental technicians responded to the questionnaire (response rate 20%). Their demographic variables are summarised in Table 2. The most common region for dental technician participants to work in was Auckland (34%) and then Canterbury (25%). Most technicians did more than 16 indirect restorations a month (60%). Fifty‐seven percent of dental technicians felt as if they had lost clients due to CAD/CAM related reasons with 43% feeling as if it was due to chairside systems and 27% saying it was due to international outsourcing.

**Table 2:** Demographics of dental technicians.

Option	Count	Percentage (%)
Which race/ethnicity best describes you (please select all that apply)		
New Zealand European	10	31
Māori	1	3
Chinese	5	16
Other	7	53

Please choose your work location based on region? (Please select all that apply)		
Northland	4	13
Auckland	11	34
Waikato	1	3
Hawkes Bay	2	6
Taranaki	1	3
Wellington	3	9
Canterbury	8	25
Otago	6	19
Southland	2	6

How many fixed prostheses (such as single‐unit crowns, fixed partial dentures, bridges or onlays) do you do each month in your practice?		
1–5	10	33
6–10	2	7
>16	18	60

Have you lost any regular clients over the last 5 years due to CAD/CAM‐related reasons?		
Yes as CAD/CAM has allowed dentists to send STL files long distance and outsource to overseas laboratories	8	27
Yes due to chairside CAD/CAM systems	13	43
No	13	43

Does your laboratory have a CAD/CAM system? (Scanning and milling done in your laboratory)		
No	2	7
Yes	24	80
I will not buy one (please explain why)	1	3
I only have a CAD system so scanning is done in your laboratory and then it is sent to a milling centre	2	7
If you intend purchase one in the future or used to have one (please specify which type of system)	1	3

### 3.1. CAD/CAM Users

Fifty‐nine percent of dentists categorised themselves as CAD/CAM users, and their responses are summarised in Table 3.

**Table 3:** Responses from dentists who are CAD/CAM users.

Option				Count	Percentage (%)
How long have you been using CAD/CAM for?					
0–5 years				94	53
6–10 years				38	22
11–15 years				20	11
>15 years				24	14

What precipitated your move towards a CAD/CAM workflow? (Please tick all that apply)					
To reduce lab fees				83	47
To improve standard of care				111	63
To improve productivity and time efficiency				151	86
To use new dental materials which can only be fabricated with CAD/CAM, for example, zirconia				49	28
To keep up with technology				141	80
Make communication easier				67	38
As a marketing tool for patients				42	24
Easier for auxiliaries/team				26	15
Reduce workflow stress				86	49
Reduce need for remakes				54	31
Keep up with patient demand				49	28
Other				18	10

Which aspects of the digital workflow do you use? (Please tick all that apply)					
Chairside CAD/CAM (e.g., CEREC)				115	66
Intra‐oral digital impression				162	93
Laboratory scanning of impressions or casts				42	24
Computer‐aided design (CAD by laboratory or specialist milling centre)				87	50
Computer‐aided manufacturing (CAM by laboratory or specialist milling centre)				85	49
Other				6	3

How are your fixed prostheses manufactured? (Please tick all that apply)					
Milled in‐house				113	65
Sent to local lab within same city/town				100	57
Sent to local lab from different city				70	40
Sent to an international lab				27	16

What percentage of indirect restorations are done through a chairside digital workflow like CEREC, 3shape?					
Less than 25%				13	12
25%–50%				9	8
51%–75%				16	14
76%–99%				52	46
100%				22	20

How frequently do you use these chairside technologies?					
Daily				45	40
Weekly				28	25
Several times a week				26	23
Monthly				11	10
Rarely				2	2

Where did you undertake your CAD/CAM system training? (Please tick all that apply)					
Companies providing CAD/CAM system				104	60
Private courses within NZ				80	46
Private courses overseas				41	24
Self‐taught or taught by other user, etc.				116	67
Dental school				32	18
Other				12	7

What percentage of the time do you use any type of digital workflow for fixed prostheses over a conventional workflow?					
Less than 25%				24	14
25%–50%				14	8
51%–75%				20	12
76%–99%				67	39
100%				48	28

**Rate your level of agreement with each statement**	**Strongly disagree**	**Disagree**	**Neutral**	**Agree**	**Strongly agree**

Appointments for the digital impression of a CAD/CAM crown take longer than those for conventional crowns	36 (30%)	52 (43%)	21 (18%)	9 (8%)	2 (2%)
Appointments for the insertion of a CAD/CAM crown take longer than conventional crowns	26 (21%)	52 (43%)	37 (31%)	4 (3%)	2 (2%)
My CAD/CAM training was sufficient	9 (5%)	23 (14%)	45 (27%)	68 (41%)	22 (13%)
CAD/CAM led to changes in my use of dental materials	6 (4%)	24 (14%)	24 (14%)	72 (43%)	40 (24%)

Many dentists used multiple forms of CAD/CAM. The most used types of CAD/CAM were intraoral digital impressions (93%), followed by chairside restoration systems (66%), computer‐aided design (50%), computer‐aided manufacture (49%), then laboratory scanning of impressions (24%). This meant 55% of total dentists were using intraoral scanners and 39% were using chairside restoration systems.

Eighty percent of dental technicians indicated that they were CAD/CAM users, while 7% only used CAD and sent restorations to be manufactured in milling centres. Their results are summarised in Table 4. Seventy‐one percent of dental technicians use CAD/CAM for most of their restorations. Eighty‐three percent of dental technicians started using CAD/CAM within the last 10 years.

**Table 4:** Responses from dental technicians who were CAD or CAD/CAM users.

Option	Count	Percentage (%)
How long have you been using CAD/CAM for?		
0–5 years	6	25
6–10 years	14	58
>15 years	4	17

Approximately what percentage of your permanent restorations are done through the CAD/CAM system?		
10%–25%	2	8
25%–50%	1	4
51%–75%	4	17
76%–100%	17	71

Does your laboratory have a CAD system? (Scanning is done in your laboratory and then it is sent to a milling centre)		
No	2	8
Yes	21	84
I intend to purchase one in the future	1	4
If you intend to purchase one in the future or used to have one (please specify which type of system)	1	4

After scanning and designing, who manufactures your restorations designed through CAD?		
In‐house	22	100
Milling centre (please specify what type of restorations)	3	14
Milling laboratory (please specify what type of restorations)	4	18

What precipitated your move towards a CAD/CAM workflow? (Please tick all that apply)		
As a marketing tool	6	23
Other (please specify)	3	12
There was demand from dentists to adopt	11	42
To improve communication with dentists	12	46
To improve productivity and time efficiency	22	85
To improve standard of care	16	62
To increase profit	11	42
To keep up with technology	22	85
To use new dental materials which can only be fabricated with CAD/CAM, for example, zirconia	16	62

Three quarters of dentists had started using CAD/CAM within the last 10 years. The most common methods of learning how to use CAD/CAM were being self‐taught or being taught by another user (67%), receiving training from the companies providing the CAD/CAM system (60%), private courses within NZ (46%), private courses overseas (24%) and then from dental school (18%). Despite most CAD/CAM users having received some form of training only 54% felt as if their CAD/CAM training was sufficient.

The main factors that facilitated CAD/CAM adoption by dentists and dental technicians were improving productivity and efficiency, keeping up with technology, and improving standard of care. Sixty‐seven percent of dentists and 81% of dental technicians felt as if CAD/CAM had led to a change in their use of dental materials.

Seventy‐one percent of dental technicians did over three quarters of their indirect restorations through a CAD/CAM system. When dentists compared digital workflow and conventional workflow appointment lengths for impressions and insertion CAD/CAM users did not tend to feel as if the digital workflow was slower.

### 3.2. Non‐CAD/CAM Users

Thirty‐one percent of dentists were non CAD/CAM users and 10% were past CAD/CAM users. Their results are summarised in Table 5.

**Table 5:** Responses from dentists who are non‐CAD/CAM users

Option				Count	Percentage (%)
Why do you not use CAD/CAM? (Please tick all that apply)					
High costs				47	48
Inferior quality of restorations				12	12
Lack of sufficient training or expertise				34	35
General lack of familiarity with technology				32	33
Do not see that there are any advantages over conventional techniques				20	20
Equipment maintenance or reliability concerns				21	21
Limited patient demand for CAD/CAM restorations				19	19
Lack of support or technical assistance				14	14
Other				28	29

Why did you stop using CAD/CAM? (Please tick all that apply)					
Higher costs				4	13
Inferior quality of restorations				8	27
Could not learn how to use the system				2	7
Did not see that there are any advantages over conventional techniques				5	17
Other				19	63

**Rate your level of agreement with each statement**	**Strongly disagree**	**Disagree**	**Neutral**	**Agree**	**Strongly agree**

I will likely incorporate CAD/CAM into my workflow in future practice	12 (9%)	12 (9%)	41 (32%)	41 (32%)	22 (17%)

The most common barriers for dentists and dental technicians to the adoption of CAD/CAM were high cost and lack of sufficient training. The main reasons dentists stopped using CAD/CAM were the inferior quality of restorations. Many participants also mentioned moving to a practice that no longer had the equipment as a reason for cessation of use of CAD/CAM. Half of dentists that were non‐CAD/CAM users felt as if it was likely they would incorporate it into their future workflow.

### 3.3. Communications Dentist–Dental Technician

The responses that relate to prescriptions are summarised in Table 6. The most frequently included details in prescriptions were material type (97%) and ceramic shade (95%). Details relating to the workflow and fabrication method were less common with only 35% of dentists regularly indicating whether they wanted a digital workflow and 27% listing the fabrication method.

**Table 6:** Responses on laboratory prescriptions.

Dentists					
Option				Count	Percentage (%)
Do you frequently work with dental technicians or laboratories for fixed prostheses?					
Very often				151	52
Often				59	20
Sometimes				53	18
Rarely				27	9
Never				3	1

Who takes the leading role in treatment planning and prosthetic design (initiative with regard to prostheses)?					
Dentists mainly exercise initiative				111	39
Technicians mainly exercise initiative				13	5
Technicians are often consulted when it comes to specific cases and parts				67	23
Decisions are made in collaboration with each other				96	33

What details do you put in your fixed prosthesis prescriptions? (Please tick all that apply)					
Type of material, for example, zirconia or lithium disilicate				278	97
Ceramic shade				271	95
Type of margin				109	38
Type of workflow, for example, digital or conventional				100	35
Method of ceramic fabrication, for example, pressed or milled				78	27
Method of coping fabrication, for example, printing or conventional wax up				35	12
Other				20	7

Dental technicians					

**Rate your level of agreement with each statement**	**Strongly disagree**	**Disagree**	**Neutral**	**Agree**	**Strongly agree**

CAD/CAM had changed my use of dental materials	2 (7%)	0	4 (15%)	10 (37%)	11 (41%)
I often contact the dentist to change the material or design because of the availability of digital technologies	1 (4%)	8 (30%)	10 (37%)	3 (11%)	5 (19%)
Information in the prescription is more commonly missing when dentists send impressions and models as optical scans over physical models and impressions	2 (8%)	6 (23%)	12 (46%)	5 (19%)	1 (4%)

**Option**				**Count**	**Percentage**

Who takes the leading role in treatment planning and prosthetic design (initiative with regard to fixed prostheses)?					
Decisions are made in collaboration with each other				12	44%
Dentists mainly exercise initiative				4	15%
Technicians are often consulted when it comes to specific cases and parts				7	26%
Technicians mainly exercise initiative				4	15%
**Rate your level of agreement with each statement**	**Strongly disagree**	**Disagree**	**Neutral**	**Agree**	**Strongly agree**

Prescriptions for fixed prostheses are usually complete enough for you to provide your best service	0	3 (11%)	9 (33%)	10 (37%)	5 (19%)
Prescriptions for fixed prostheses usually contain only the minimum amount of information necessary to get the job done	3 (11%)	6 (22%)	6 (22%)	11 (41%)	1 (4%)
Prescriptions for fixed prostheses usually require a call to the dentist to get more information	1 (4%)	9 (33%)	8 (30%)	9 (33%)	0
Prescriptions for fixed prostheses usually lack in customisation or personalisation	3 (11%)	10 (37%)	8 (30%)	5 (19%)	1 (4%)

**Option**				**Count**	**Percentage (%)**

What details are usually missing in fixed prosthesis prescriptions?					
Type of workflow, for example, digital or conventional				7	27
Type of material, for example, zirconia or lithium disilicate				6	23
Type of margin				11	42
Other (please specify)				6	23

Dental technicians listed certain details as regularly missing in lab prescriptions for indirect restorations. The type of margin was the most common with 42% saying it was usually missing. Twenty‐seven percent said type of workflow, and 23% said type of material.

Bivariate analyses found an association between being a CAD/CAM user and having a large or small group practice, being a male dentist, and doing a greater number of indirect restorations. Additionally, an association was found between fewer years since graduation and feeling as if your CAD/CAM training was sufficient. This is described in Table 7.

**Table 7:** Bivariate analyses.

Sociodemographic factor	Yes		No		Used in the past but not now		Chi‐square	
	*N*	%	*N*	%	*N*	%	χ^2^	*p*
Gender								
Male	73	67	24	22	12	11	9.551	0.049^a^
Female	57	50	45	40	10	9		
Missing	37	51	27	38	8	11		
Total	167		96		30			

Ethnicity								
NZ European	87	59	41	28	19	13	18.171	0.111
Māori	1	33	2	67	0	0		
Indian	14	58	8	33	2	8		
Chinese	31	60	17	33	3	6		
NZ European and others	3	21	9	64	2	14		
Other	31	60	18	35	3	6		

Region								
Auckland	64	65	25	26	9	9	32.622	0.173
Bay of Plenty	10	67	4	27	1	7		
Canterbury	17	50	14	41	3	9		
Gisborne	1	50	1	50	0	0		
Hawkes Bay	3	38	4	50	1	13		
Manawatu Whanganui	7	78	0	0	2	22		
Nelson–Marlborough	8	53	5	33	2	13		
Northland	6	67	3	33	0	0		
Otago	12	41	11	38	6	21		
Southland	1	14	4	57	2	29		
Taranaki	1	25	3	75	0	0		
Waikato	11	52	9	43	1	48		
Wellington	26	65	11	28	3	8		
Westcoast	0	0	2	100	0	0		

Number of indirect restorations per month								
1–5	49	41	53	45	17	14	25.280	<0.001^a^
6–10	53	63	23	27	8	10		
11–15	33	77	6	14	4	9		
>16	32	68	14	30	1	2		

Qualification								
GDP	136	55	84	34	28	11	3.666	0.160
Postgrad	31	69	12	27	2	4		

Practice type								
Franchise	9	36	12	48	4	16	13.953	0.03^a^
Large group practice	76	68	25	22	11	10		
Public hospital	4	36	6	55	1	9		
Small solo practice	78	54	53	37	14	10		

Independent samples Kruskal–Wallis test								
Question compared to years since graduation						*X* ^2^	*d*	*p*
Appointments for the digital impression of a CAD/CAM crown take longer than those for conventional crowns						3.321	4	0.506
Appointments for the insertion of a CAD/CAM crown take longer than conventional crowns						8.290	4	0.082
My CAD/CAM training was sufficient						12. 983	4	0.011^a^
CAD/CAM led to changes in my use of dental materials						2.893	4	0.576

^a^ *p* value < 0.05. Statistically significant results.

## 4. Discussion

This study provides one of the largest surveys to date on the adoption of digital technologies for indirect restorations in NZ, capturing responses from over 300 dentists and 40 dental technicians. While the sample represents a substantial proportion of the national workforce and offers good statistical precision, some limitations must be acknowledged. Participation was voluntary, raising the possibility of self‐selection bias and the cross‐sectional design captures current practice but not changes over time. The response rate among dentists was 19%, which introduces the potential for non‐response bias. Dentists with greater interest in digital technologies may have been more likely to participate; therefore, the prevalence figures reported in this study should be interpreted as indicative rather than definitive estimates of national adoption. Future studies could further strengthen external validity by directly comparing respondent demographics with national workforce statistics published by the Dental Council of New Zealand.

In addition, subgroup analyses such as ethnicity or practice type were constrained by smaller numbers. The number of technicians was also lower, and because registration is not mandatory in NZ, the overall coverage of the technician workforce cannot be fully determined. In addition, all data were self‐reported by participants. Therefore, variables such as workflow usage, restoration volume and perceptions of efficiency or quality reflect professional perceptions rather than objectively measured clinical outcomes. Despite these limitations, the study provides important insights into current adoption patterns, training experiences and communication between dentists and technicians.

CAD/CAM use among NZ dentists appears to be increasing; in 2016, 32% reported doing chairside indirect restorations compared with 39% in this survey [9]. Technicians have increased at a greater rate bringing NZ closer to international equivalents [19]. As of 2012, 25% of laboratories had CAD systems and only 7.9% had full CAD/CAM systems, whereas now, 80% of technicians have full CAD/CAM systems and 7% of the remainder have CAD systems [18]. In 2012, only half of technicians felt CAD/CAM resulted in less clients, which has increased to 57% in this survey [18].

CAD/CAM training is likely inadequate as only 54% of dentists felt as if their training was sufficient and lack of training was a common barrier. Dental school was not a common method of learning, which highlights how previously CAD/CAM was excluded from foundational teaching. These findings also highlight implications for dental education and continuing professional development. As digital technologies become increasingly integrated into restorative workflows, both undergraduate training and targeted courses for practising dentists may be important to support confidence and competence in CAD/CAM use.

Recent graduates were more likely to feel as if their CAD/CAM training was sufficient, which translates to recent improvements in CAD/CAM training within dental schools [21]. For older dentists, however, CAD/CAM would have only become available after graduation, so an active choice is required to pursue training. They also were likely exposed to poorer historic materials that encouraged a negative view of CAD/CAM, and many are deterred by the need for training as they do not believe it is worth investing the limited time left in their careers [22, 23]. To increase CAD/CAM usage the training of those already working could be improved through external courses and targeted training [14, 24].

Quality is often the key driver overseas, which contrasts with NZ research, which usually highlights efficiency [13, 14]. Current users often list quality as a reason for adopting and past users often believe it produces inferior restorations. Its prominence on both sides of decision‐making highlights the importance of perceived quality. However, it should be noted that this study assessed clinicians’ perceptions rather than objective clinical outcomes. More long‐term research is required to decide which workflow is superior; however, patient and clinician factors are believed to be more important in determining restoration quality [25–28].

High costs from the initial investment and maintenance are a consistent barrier to CAD/CAM adoption [14, 29, 30]. Contrastingly, many CAD/CAM users in this survey cited that avoiding laboratory fees was a driver to adoption. Both workflows have costs associated so individual perception likely plays a significant role in which costs dentists believe are more significant.

The association between practice type and CAD/CAM usage is consistent with the literature [13, 14]. The lack of CAD/CAM in public practice could relate to limited funding or lower prioritisation of indirect restorations. The reasons for the differences in uptake between group practices and corporate practices is unknown. Corporations may be less willing to invest money, whereas group practice dentists have more independence to purchase technology they are interested in.

In treatment planning technicians were more likely to say decisions were made collaboratively, whereas dentists usually felt as if they exercised initiative. This difference could be attributed to dentists limited awareness of the decisions that technicians are making. As details are commonly missed in the prescription, technicians frequently need to make decisions to compensate. These findings differ to Japan where technicians usually felt that dentists made most of the decisions [20].

The poor quality of many laboratory prescriptions was a significant theme and highlighted the need for better prescription writing. Only half of technicians felt prescriptions usually included enough detail, which echoes international research [17]. Thirty‐three percent of NZ technicians also often had to contact dentists for more information. There was varying agreement on the details commonly present in prescriptions. Twenty‐seven percent of technicians felt workflow type was regularly missing, but only 35% of dentists regularly included it. This could potentially be because technicians feel this detail is unnecessary as they would rather decide or their laboratory only offers one workflow. Both agreed dentists consistently included shade. However, answers on material type differed. Ninety‐seven percent of dentists said they regularly included material type whereas 23% of technicians felt it was regularly missing. There may be different standards on the detail required for material description. It has previously been reported that some dentists only provide the brand, unaware of the variety within brands [31].

Poor communication of material selection can lead to unfavourable results. Research consistently finds technicians often must call up dentists to gain more clarity [16]. Beyond efficiency issues, lack of clarity in the material choice can contribute to inappropriate material selection as it leaves a wide margin for error [31]. This can result in restorations that do not meet patient standards. To reduce inefficiencies, improved prescriptions are necessary for clear communication.

Both dentists and technicians agreed that CAD/CAM had influenced their material selection, which is consistent with overseas research [14]. Sixty‐two percent of technicians listed new materials as a driver, whereas only 28% of dentists agreed. This difference could be because dentists can offload their demands for materials that require CAD/CAM through prescriptions to laboratories. This would imply that dentists are putting pressure on technicians, which is supported by the 42% of technicians that indicated dentists had encouraged CAD/CAM usage.

There are several possible explanations for why CAD/CAM users reported completing more indirect restorations per week. One possibility is that digital workflows improve efficiency and productivity, allowing clinicians to complete more procedures. Alternatively, dentists who already perform a higher volume of indirect restorations may be more likely to adopt CAD/CAM technologies in order to streamline their workflow. Because this study used a cross‐sectional design, the direction of this relationship cannot be determined.

## 5. Conclusions

This study highlights the growing role of CAD/CAM technology in dentistry, with dental technicians adopting it faster than dentists. Cost and lack of training remain key barriers. Addressing these through improved education and targeted training is essential for wider clinical integration.

## Author Contributions

All authors made significant contributions to this research. Ana Kahurangi Conway Downey conducted the survey and drafted the manuscript. Arthi Veerasamy developed the study concept, ensured questionnaire validity, and analysed the data. Arthi Veerasamy and Vincent Bennani supervised the project.

## Funding

This work was funded by the New Zealand Dental Association (Grant CF139529). Open access publishing facilitated by University of Otago, as part of the Wiley ‐ University of Otago agreement via the Council of Australasian University Librarians.

## Conflicts of Interest

The authors declare no conflicts of interest.

## Data Availability

The data that support the findings of this study are available upon request from the corresponding author. The data are not publicly available due to privacy or ethical restrictions.
